# Localization of Receptor Site on Insect Sodium Channel for Depressant β-toxin BmK IT2

**DOI:** 10.1371/journal.pone.0014510

**Published:** 2011-01-14

**Authors:** Huiqiong He, Zhirui Liu, Bangqian Dong, Jianwei Zhang, Xueqin Shu, Jingjing Zhou, Yonghua Ji

**Affiliations:** 1 Lab of Neuropharmacology and Neurotoxicology, Shanghai University, Shanghai, People's Republic of China; 2 Graduate School of Chinese Academy of Sciences, Shanghai Institute of Physiology, Shanghai Institute of Biological Sciences, Chinese Academy of Sciences, Shanghai, People's Republic of China; University of Southampton, United Kingdom

## Abstract

**Background:**

BmK IT2 is regarded as a receptor site-4 modulator of sodium channels with depressant insect toxicity. It also displays anti-nociceptive and anti-convulsant activities in rat models. In this study, the potency and efficacy of BmK IT2 were for the first time assessed and compared among four sodium channel isoforms expressed in *Xenopus* oocytes. Combined with molecular approach, the receptor site of BmK IT2 was further localized.

**Principal Findings:**

2 µM BmK IT2 strongly shifted the activation of DmNa_v_1, the sodium channel from *Drosophila*, to more hyperpolarized potentials; whereas it hardly affected the gating properties of rNa_v_1.2, rNa_v_1.3 and mNa_v_1.6, three mammalian central neuronal sodium channel subtypes. (1) Mutations of Glu_896_, Leu_899_, Gly_904_ in extracellular loop Domain II S3–S4 of DmNa_v_1 abolished the functional action of BmK IT2. (2) BmK IT2-preference for DmNa_v_1 could be conferred by Domain III. Analysis of subsequent DmNa_v_1 mutants highlighted the residues in Domain III pore loop, esp. Ile_1529_ was critical for recognition and binding of BmK IT2.

**Conclusions/Significance:**

In this study, BmK IT2 displayed total insect-selectivity. Two binding regions, comprising domains II and III of DmNa_v_1, play separated but indispensable roles in the interaction with BmK IT2. The insensitivity of Na_v_1.2, Na_v_1.3 and Na_v_1.6 to BmK IT2 suggests other isoforms or mechanism might be involved in the suppressive activity of BmK IT2 in rat pathological models.

## Introduction

Voltage-gated sodium channels (VGSC) are key membrane proteins responsible for neuron excitability, consisting of an ion-conducting α-subunit accompanied by one or more auxiliary subunits [1]. Generally, the α-subunit comprising four repeated domains (DI–DIV), each containing six transmembrane α-helixes (S1–S6) and a hairpin-like pore loop between S5 and S6 [2], split into an N-terminal part (SS1) and a C-terminal part (SS2). Despite the high structure similarity, various VGSC subtypes display distinct distribution, gating properties and function activities. Some neurotoxins can differentiate among them with preference for certain subtype(s) [3], thus providing clues about the structure-fuction relationship of VGSCs and a potential molecule library for novel drug design or insecticide development.

Amongst the neurotoxins purified from scorpions, β-toxins shift the voltage dependence of VGSC activation to cause subthreshold channel opening, which can be enhanced when channels are preactivated by a depolarizing prepulse [4]. According to the phyletic-bioactivity, the β-toxins may be further divided into: β-mammal toxins, depressant or excitatory insect-specific β-toxins and TsVII-like toxins acting on both mammals and insects [3]. The group of β-toxins is deemed to bind to a common receptor site-4 on VGSC α-subunits, which, however, shows a rather complex picture. The binding sites for β-mammal toxin CssIV (from *Centruroides suffusus suffusus*) and TsVII-like toxin Tz1 (from *Tityus zulianus*) have been mapped to DII S1–S2, DII S3–S4 and DIII SS2-S6 on mammalian VGSCs [4], [5], [6], [7]. The effect of TsVII (i.e. Tsγ from *Tityus serrulatus*) on reducing peak currents are also conferred by the S4 segments of DIII and DIV [8], [9]. The excitatory and depressant β-toxins act distinctly, though they both target insect VGSCs [10], [11]. DII of DmNa_v_1 is implicated in the selective recognition of excitatory toxin AahIT (from *Androctonus australis hector*) [12], while several channel regions (DI S5-SS1, DI SS2-S6, DIII SS2-S6, and DIV SS2-S6) may be involved in the interacion with depressant toxin LqhIT2 (from *Leriurus quinquestriatus hebraeus*) [11]. Based on the results of mutation experiments applied on rat VGSCs and information provided by structure analysis of LqhIT2 [13], [14], some possible interaction spots in DII S3–S4 of DmNa_v_1 were deduced. However, no site-directed mutagenesis has been performed on insect VGSC yet as to dissect the receptor site for depressant β-toxins.

BmK IT2, a depressant β-toxin from the scorpion *Buthus martensi* Karsch, can induce strong insect toxicity [15]. Like other depressant toxins, such as LqhIT2 [11], [16], BmK IT2 possesses two non-interacting binding sites (the high/low-affinity binding sites) on insect nerve membranes [17], [18]. Despite typical anti-insect features of depressant β-toxins, BmK IT2 displayed antinociceptive and anticonvulsant activities in rat models [19], which were attributed to the specific modulation on brain VGSCs [20]. Such effects against mammals have also been observed in other depressant β-toxins [21], [22], [23] and explained as a consequence of adaptive evolution of these toxins. However, the binding affinity of BmK IT2 to rat brain synaptosomes was quite low [17], [18] and the specific target is still unidentified.

To forward the understanding for the binding features of depressant β-toxins and their intriguing functional diversity, in the present study, we attempted to address the following issues: 1) Can BmK IT2 modulate the mammalian VGSC subtypes from central neuronal system (i.e. Na_v_1.2, Na_v_1.3 and Na_v_1.6)? 2) What is the selectivity of BmK IT2 between these mammal subtypes and insect VGSC DmNa_v_1? 3) What is the binding/recognition site on insect VGSC for BmK IT2?

## Materials and Methods

### Materials

BmK IT2 was purified by column chromatography from the crude venom of the Asian scorpion *Buthus martensi* Karsch as described previously [15]. The purity of the toxin was confirmed by mass spectrometry.

The genes encoding the sodium channel α-subunit DmNa_v_1 (P35500.3) from *Drosophila* paralytic temperature-sensitive and the auxiliary TipE subunit were kindly provided by J. Warmke (Merck, New Jersey, USA) and M. S. Williamson (IACR-Rothamsted, UK), respectively. Plasmids in combination with cDNAs of rat/mouse VGSC α-isoforms i.e. rNa_v_1.2 (CAA27287), rNa_v_1.3 (CAA68735) and mNa_v_1.6 (Q9WTU3.1), as well as β1 subunit were originally from Dr. Alan L. Goldin (University of California, USA).

### Construction of channel chimeras and mutants

Five endogenous restriction sites in rNa_v_1.2α ORF were used to excise DNA fragments coding for the four channel domains (DI: XhoI/XmaI, DII: XmaI/BglII, DIII: BglII/BstEII, DIV: BstEII/PacI). The parallel DNA fragments of DmNa_v_1 corresponding to the four parts were amplified by PCR with primers containing restriction sites for XhoI, XmaI, BglII, BstEII and PacI in homologous positions. Four chimeric channels (ChD1, ChD2, ChD3 and ChD4) were generated by introducing four DmNa_v_1 fragments into excised rNa_v_1.2α so that individual domains of rNa_v_1.2α were replaced by those of DmNa_v_1 channel. The exchange of DIII SS2 loops between DmNa_v_1 and rNa_v_1.2α were accomplished with PCR-based mutagenesis, giving rise to two products: L(Dm)Na_v_1.2 (M_1425_I, D_1426_Q, Y_1429_N, A_1430_D, V_1432_I, N_1436_E, E_1438_D, L_1439_K, K_1442_I, Y_1443_R, D_1445_T) and L(1.2)DmNa_v_1 (I_1512_M, Q_1513_D, N_1516_Y, D_1517_A, I_1519_V, E_1523_N, D_1525_E, K_1526_L, T_1532_D). L(Dm)Na_v_1.2 means that the loop from the DmNa_v_1 as donor was constructed in rNa_v_1.2 as acceptor and vice versa.

In addition, site-directed mutagenesis was performed to introduce a series of mutations into DmNa_v_1 and the resulting mutants were as follows: multiple-residue mutant DmM5 (I_1512_M/Q_1513_D/N_1516_Y/D_1517_A/I_1519_V), double-residue mutant DmI_1529_K/R_1530_Y and single-residue mutants DmD_838_C, DmE_896_C, DmL_899_C, DmG_904_N, DmE_1523_N, DmK_1526_L, DmI_1529_K and DmR_1530_Y. Primers were designed with Primer5.0 (PremierBiosoft, USA) (See Table S1). All clones were verified by DNA sequencing according to their wild-type sequences (See Figure S1). Plasmid DNAs were harvested and isolated from XL1-blue E. coli (Stratagene, USA).

### Voltage-gated sodium channel expression and Electrophysiological studies

Mammalian VGSCs rNa_v_1.2α, rNa_v_1.3α and mNa_v_1.6α were expressed in *Xenopus* oocytes accompanied with auxiliary subunit β1 while the insect VGSC DmNa_v_1 was coexpressed with TipE for generating robust Na^+^ currents.

The genes for wild-type VGSCs, chimeras, mutants and those for the auxiliary subunits (TipE and β1) were transcribed *in vitro* using T7 RNA-polymerase and the mMESSAGE mMACHINE™ system (Ambion, Austin, TX). *Xenopus* laevis oocytes were prepared [24] and injected with 0.5–10 ng of each wild-type cRNA species (1∶1 weight mixture respectively for rNa_v_1.2α/β1, rNa_v_1.3α/β1, mNa_v_1.6α/β1 and Para/TipE) or with 35–50 ng of each cRNA from chimeric/mutant channels with β1/TipE cRNA (1∶1 weight ratio). Oocytes were incubated at 20°C for 2–5 days in ND96 solution (in mM: NaCl 96, KCl 2, CaCl_2_ 1.8, MgCl_2_ 2 and HEPES 5, pH 7.5), supplemented with 5 mM pyruvate and 0.1 mg/ml gentamicin.

Two-electrode voltage-clamp recordings were performed at room temperature (18°–22°C) using the TURBO TEC-03X amplifier (npi Instruments, Germany) and Cellwork E5.5 software (npi electronic Instruments). Voltage and currents electrodes were filled with 3 M KCl. Currents were filtered at 1.3 kHz and sampled at 10 kHz with a four-pole Bessel filter. Bath solution composition was (in mM): NaCl 96, KCl 2, CaCl_2_ 1.8, MgCl_2_ 2 and HEPES 5 (pH 7.4). Toxin BmK IT2 were diluted with bath solution and applied directly to the bath at desired concentration.

From a holding potential of −100 mV, oocytes were depolarized with a three-step protocol [25]. The first and last test depolarization of 25 ms duration ranged from −70 mV to +70 mV in steps of 10 mV. The second depolarization (PP) to a voltage of −10 mV was used to prime the channels ensuring maximal binding of the β-toxin to the channel. The third segment of 25 ms at −120 mV ensured recovery from inactivation. Repetition interval was 2 seconds. The peak currents elicited in the test depolarizations were plotted as a function of voltage, resulting in current/conductance-voltage relationships (I/G–V curves). This approach provided an assessment of the BmK IT2 effect on channel activation with and without a depolarizing prepulse (PP) in one experiment.

### Data analysis

Data were acquired by Cellworks Reader 3.6 (NPI electronic Instruments) and analyzed with Origin 7.5 (Northampton, USA) software.

Only recordings with leakage below 0.10 µA and fluctuation within 0.05 µA were selected in statistical analysis. The results are shown as means ± SEM with the number of experiments provided as *n* in the talble legends.

Mean conductance (G) was calculated from peak current/voltage relations using the equation G = I/(V−V_rev_), where I is the peak current elicited upon depolarization, V is the membrane potential, and V_rev_ is the reversal potential. The voltage dependence for the activation of I was fit with the Boltzmann relation, G/G_max_ = 1/[1+exp(V_1/2_−V)/*k*_m_], where V_1/2_ is the voltage for half-maximum activation and *k*_m_ is the slope factor. The EC_50_ values were determined by measuring the currents induced by BmK IT2 at the voltage of channel activation threshold (−40 mV).

## Results

### Efficacy of BmK IT2 on VGSC isoforms from insect and mammalian central neuronal system

**Figure 1:**
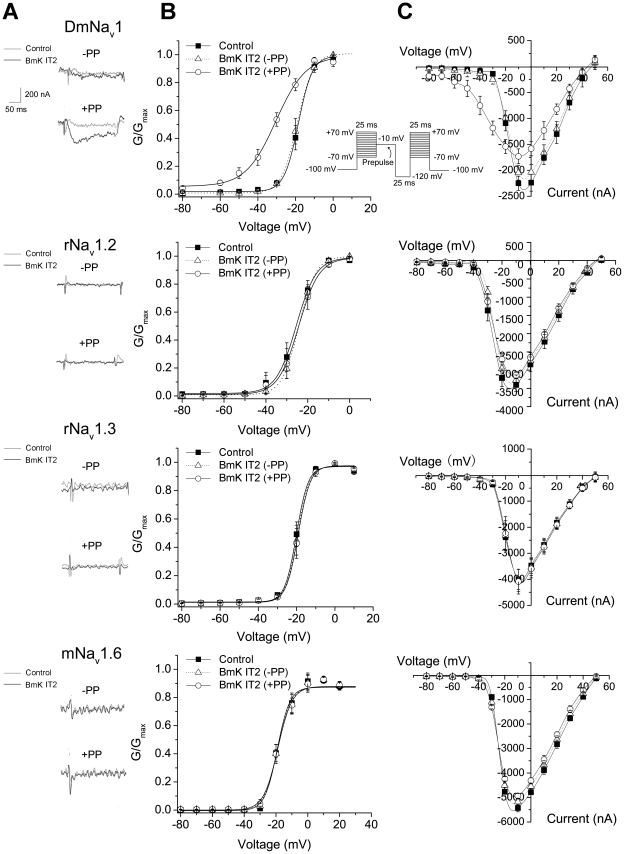
Effect of BmK IT2 on wild-type VGSCs expressed in *Xenopus* oocytes. A. Current responses of rNa_v_1.2, rNa_v_1.3, mNa_v_1.6 and DmNa_v_1 channels to a test voltage of −50 mV, where channels were closed under control conditions (gray traces). Black traces represented currents in the presence of 2 µM BmK IT2 without a prepulse (−PP, upper panel) and with a prepulse (+PP, lower panel). The scale bar in figure 1A covered all four embodied currents. B. Normalized conductance plotted as a function of voltage for the indicated channel subtypes. C. Current-voltage curves for the indicated channel types. ▪, control conditions; ▵, 2 µM BmK IT2 without a prepulse (−PP); ○, 2 µM BmK IT2 with a prepulse (+PP).

Using the two-electrode voltage clamp recording, BmK IT2 was subjected to a comparative study for the effects on four VGSC subtypes, rNa_v_1.2/β1, rNa_v_1.3/β1, mNa_v_1.6/β1 and DmNa_v_1/TipE expressed in *Xenopus* oocytes (Fig. 1). The voltage-dependent channel activation was investigated by a three-step protocol (see *Materials and Methods*). 2 µM BmK IT2 induced significant subthreshold currents (at −50 mV) in DmNa_v_1/TipE channels with a depolarizing prepulse (PP) of 25 ms (Fig. 1A). The half-maximal activation voltage (V_1/2_) of DmNa_v_1/TipE was shifted by about −11 mV and the slope factor (*k*_m_) was increased from 3.72 to 7.97 mV (p<0.001, n = 10) by 2 µM BmK IT2 (EC_50_ = 2.9±0.36 µM, Table 1). This shift was also observed only in the presence of a prepulse (Fig. 1B–C). In contrast, rNa_v_1.2/β1, rNa_v_1.3/β1, mNa_v_1.6/β1 were totally insensitive to BmK IT2 at concentrations of 2 µM (Fig. 1A and B) and even up to 20 µM (Fig. S2). Prolonging the PP duration to 50 ms was unable to enhance the efficacy of BmK IT2 (data not shown). Though the activation of rNa_v_1.3/β1 eventually responded to BmK IT2 at a rather high concentration (50 µM; ΔV_1/2_ = −4.84 mV, data not shown), rNa_v_1.2/β1 and mNa_v_1.6/β1 still remained insensitive. Whether or not β1 subunit was coexpressed with these mammalian VGSC subtypes did not influence the action of BmK IT2 (not shown).

**Table 1:** EC_50_ values (µM) of BmK IT2 on wild-type and chimeric/mutated VGSCs.

Channels	EC_50_ (µM)	*n*
rNa_v_1.2	>50	3
rNa_v_1.3	>20	4
mNa_v_1.6	>50	3
DmNa_v_1	2.9±0.36	5
ChD1	>50	3
ChD2	>50	3
ChD3	22.5±6.65	3
ChD4	>50	3
DmD_838_C	3.6±0.90	3
DmE_896_C	>35	3
DmL_899_C	>35	3
DmG_904_N	>50	3
L(Dm)Na_v_1.2	ND	/
L(1.2)DmNa_v_1	ND	/
DmI_1529_K/R_1530_Y	ND	/
DmM5	ND	/
DmE_1523_N	2.4±0.46	3
DmD_1525_E	3.3±0.73	3
DmK_1526_L	ND	/
DmI_1529_K	15.6±3.60	3
DmR_1530_Y	ND	/
DmT_1532_D	ND	/
DmI_1534_L	ND	/

EC_50_ values (µM) were determined as described in *Methods*. The data were represented as the mean ± SEM and *n* is the number of independent experiments.ND, not determined; /, null.

On all investigated VGSC subtypes, a small depression of current amplitude was observed (<10% for mammalian VGSCs and ∼20% for DmNa_v_1/TipE, Fig. 1C) after application of BmK IT2. There were no significant BmK IT2-induced changes in inactivation process of channels (data not shown). The results suggest that BmK IT2 exhibited distinguished subtype selectivity on sodium channels, preferring the insect target rather than mammalian central neuronal isoforms.

### Mutations in DII S3–S4 impacted BmK IT2 function on insect VGSC

**Figure 2:**
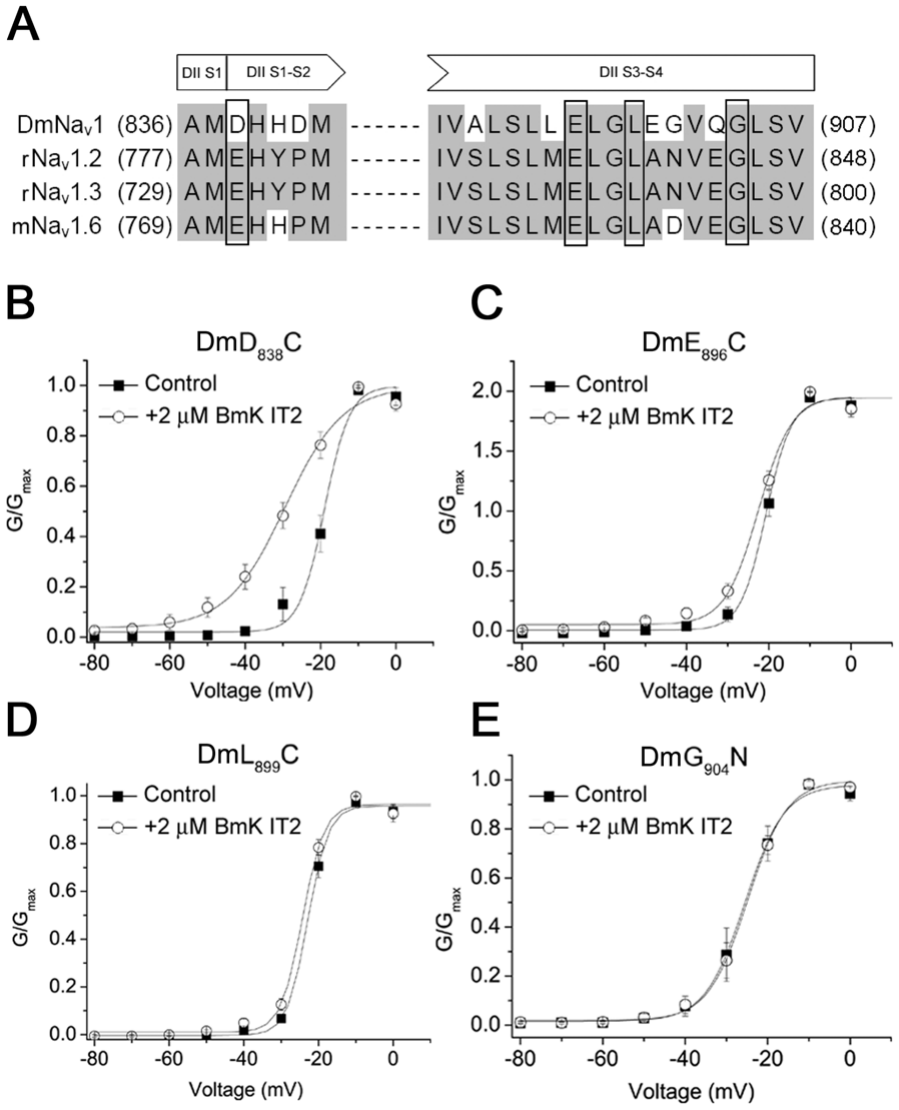
Analysis of mutations in DII of DmNa_v_1. A. Sequence comparison of extracellular loops DII S1–S2 and DII S3–S4 among wild-type VGSCs. B-E. Normalized conductance-voltage (G–V) curves of DmNa_v_1 mutants DmD_838_C, DmE_896_C, DmL_899_C and DmG_904_N in the absence (▪) and presence (○) of 2 µM BmK IT2. All the currents were recorded after applying a prepulse of −10 mV for 25 ms.

Previous reports demonstrated that substitutions introduced to DII (e.g. E_779_Q in DII S1–S2, and E_837_Q, L_840_C, G_845_N in DII S3–S4 of rNa_v_1.2α; G_658_N in DII S3–S4 of rNa_v_1.4) reduced the effects of the β-toxins Css4 and Tz1 [4], [5], [6], [7]. As for the case of depressant toxin, structural bioinformatics analysis deduced three analogous residues in DmNa_v_1 (E_896_, L_899_ and G_904_) might also be crucial in the interaction with LqhIT2 [13], [14]. Based on these studies and considering the high homology between LqhIT2 and BmK IT2, mutations of D_838_, E_896_, L_899_ and G_904_ (corresponding to E_779_, E_837_, L_840_ and G_845_ in rNa_v_1.2, Fig. 2A), were individually introduced into DmNa_v_1.

The mutants were co-expressed with TipE subunit ensuring the functional expression and currents were recorded in the same condition as that of wild type DmNa_v_1. The gating property of all mutants was not altered with respect to those of wild-type channels, thus the subsequent electrophysiological analysis was not “contaminated” by mutagenesis. The normalized conductance-voltage relationship of mutants were assessed in the absence and presence of 2 µM BmK IT2 with a 25 ms-PP. Mutant D_838_C showed the similar response to BmK IT2 as wild type DmNa_v_1 (Fig. 2B), whereas the mutations of Glu_896_, Leu_899_ and Gly_904_ totally abolished negative shift of voltage-dependent activation induced by 2 µM BmK IT2 (ΔV_1/2_<2.0 mV, Δ*k*_m_<1.0 mV, n = 7 or 8, Fig. 2C–E, Table 2). Besides, the mutants DmE_896_C, DmL_899_C and DmG_904_N were also resistant to BmK IT2 at higher concentrations (Talbe 1). This result verified that residues E_896_, L_899_ and G_904_ in DII S3–S4 of DmNa_v_1 play critical roles in responding to BmK IT2.

**Table 2:** Parameters for the voltage dependent activation of wild-type and chimeric/mutated VGSCs.

Channels	V_1/2_	V_1/2_(+BmK IT2)	ΔV_1/2_	*k*_m_	*k*_m_(+BmK IT2)	*n*
rNa_v_1.2	−25.45±0.67	−24.31±0.68	1.14±0.01	4.78±0.54	4.88±0.55	8
rNa_v_1.3	−20.06±0.32	−19.16±0.36	0.90±0.04	3.03±0.54	3.14±0.50	8
mNa_v_1.6	−19.14±0.72	−18.82±0.80	0.32±0.08	3.93±0.76	4.48±0.76	8
DmNa_v_1	−18.52±0.34	−30.44±0.70	−11.92±0.36	3.72±0.40	7.97±0.65	10
ChD1	−20.60±0.32	−20.65±0.28	−0.05±0.04	2.13±0.24	2.33±0.33	10
ChD2	−17.41±0.22	−18.28±0.21	−0.87±0.01	4.82±0.60	4.92±0.49	10
ChD3	−23.40±0.29	−29.04±0.29	−5.64±0.00	3.85±0.25	3.59±0.26	10
ChD4	−20.22±0.39	−21.66±0.41	−1.44±0.02	4.21±0.41	4.40±0.38	10
DmD_838_C	−18.95±0.62	−29.40±0.98	−10.45±0.36	3.33±0.76	7.68±0.88	7
DmE_896_C	−20.68±0.38	−22.54±0.52	−1.86±0.14	3.10±0.57	4.05±0.45	6
DmL_899_C	−22.89±0.38	−24.28±0.39	−1.39±0.01	2.76±0.28	2.90±0.22	7
DmG_904_N	−25.55±0.57	−24.89±0.57	0.66±0.00	4.80±0.46	4.79±0.45	8
L(Dm)Na_v_1.2	−21.01±0.31	−26.06±0.39	−5.05±0.08	3.71±0.34	4.79±0.32	7
L(1.2)DmNa_v_1	−21.87±0.64	−34.35±0.72	−12.48±0.08	4.76±0.58	5.48±0.62	9
DmI_1529_K/R_1530_Y	−24.82±0.48	−29.88±0.48	−5.06±0.00	4.85±0.39	5.05±0.46	8
DmM5	−21.68±0.35	−28.24±0.55	−6.56±0.20	2.85±0.40	5.73±0.49	6
DmE_1523_N	−25.32±0.56	−37.42±0.84	−12.10±0.28	3.90±0.40	6.89±0.75	7
DmD_1525_E	−21.32±0.34	−31.05±0.58	−9.73±0.24	3.32±0.40	6.50±0.52	6
DmK_1526_L	−21.54±0.39	−33.83±0.59	−12.29±0.20	3.70±0.40	5.71±0.51	8
DmI_1529_K	−23.10±0.17	−22.98±0.18	0.12±0.01	3.42±0.13	4.02±0.14	7
DmR_1530_Y	−20.35±0.51	−25.46±0.98	−5.11±0.47	4.03±0.56	7.69±0.85	8
DmT_1532_D	−20.57±0.36	−30.01±0.70	−9.44±0.34	3.46±0.46	7.41±0.64	6
DmI_1534_L	−24.64±0.42	−32.63±0.55	−7.99±0.13	2.96±0.23	5.90±0.45	7

The values of half-maximum activation voltage V_1/2_ and corresponding slope factor (*k*_m_) were determined in the absence and presence of 2 µM BmK IT2. Application of BmK IT2 shifted channel activation by ΔV_1/2_. The data were represented as the mean ± SEM and *n* is the number of independent experiments.

### DIII from DmNa_v_1 conferred BmK IT2 sensitivity to rNa_v_1.2

Although E_896_, L_899_ and G_904_ positively support the action of BmK IT2, sequence alignments (Fig. 2A) indicate these residues are also conserved in corresponding positions of all BmK IT2-insensitive mammalian VGSCs investigated. It appears that they are necessary, but not sufficient to fulfill the interaction with BmK IT2, suggesting additional channel region(s) might be involved.

**Figure 3:**
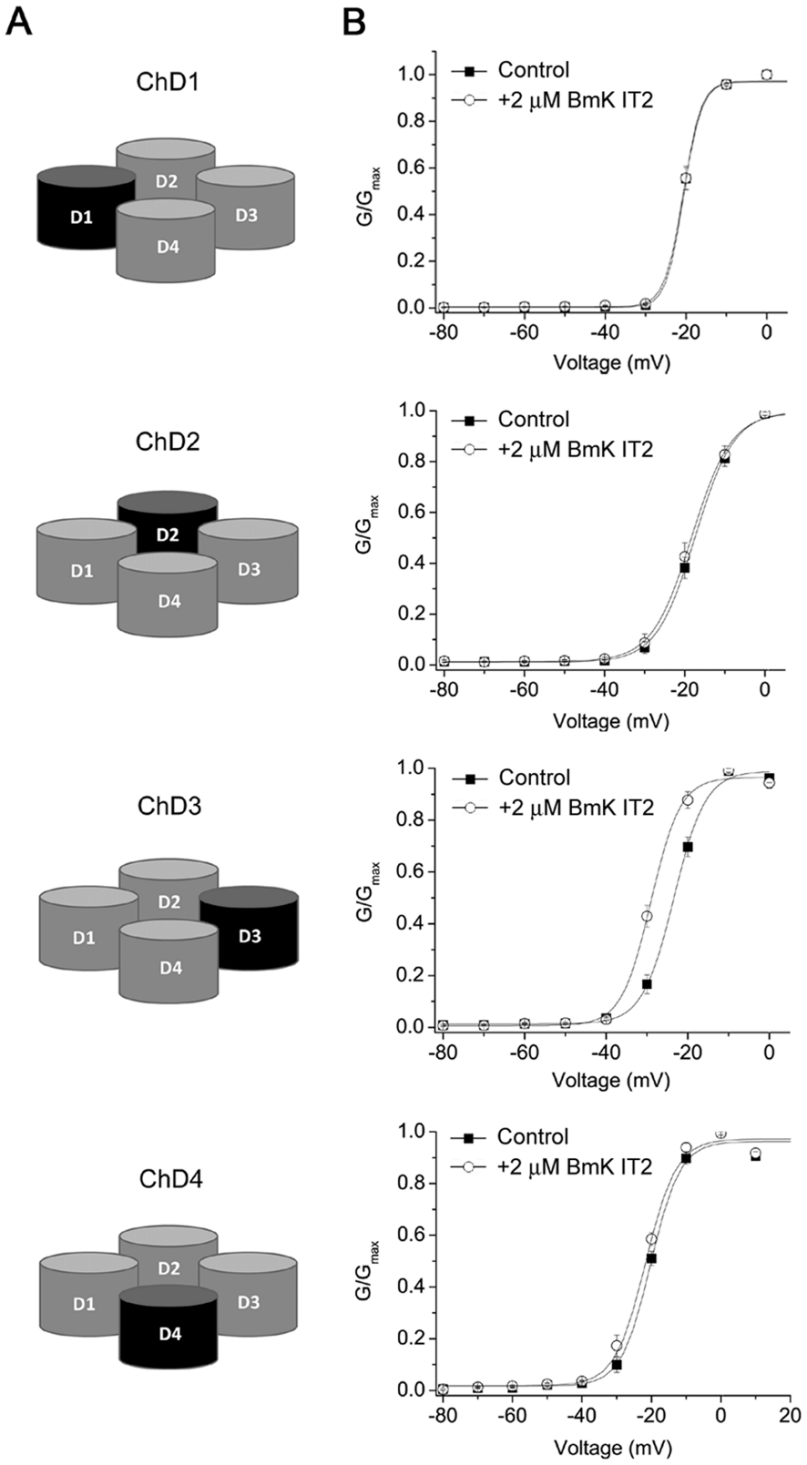
Schematic composition of DmNa_v_1-Na_v_1.2 domain chimeras and effect of BmK IT2 on four chimeric channels. A. Cartoons illustrating the construction of channel chimeras. The channel domains of rNa_v_1.2 were shown in grey, while the domains from DmNa_v_1 were shown in black. B. Normalized conductance-voltage plotted for chimeras ChD1-ChD4 before (▪) and after (○) application of 2 µM BmK IT2, with a prepulse (PP).

To find out the region(s) responsible for BmK IT2 recognition, four chimeras (ChD1, ChD2, ChD3 and ChD4; Fig. 3A) were thus constructed by replacing each domain of rNa_v_1.2α with that of DmNa_v_1 respectively. Current recordings demonstrated that the channel activities were not impaired by cross-species domain substitution. Like rNa_v_1.2α,most chimeric channels were regulated by mammalian β1 subunit but not TipE from insect (data not shown). The only exception was ChD4 that seemed insensitive to either β1 or TipE.

The activation of chimeras ChD1, ChD2 and ChD4 were hardly modified by 2 µM BmK IT2 (Fig. 3B), like wild type Na_v_1.2α/β. In contrast, ChD3 gained the response to 2 µM BmK IT2, which caused a statistically significant shift of channel activation (ΔV_1/2_ = −5.64 mV, p<0.005, n = 10) (Fig. 3B, Table 2). The increased sensitivity in ChD3 (EC_50_ = 22.5±6.65 nM, Table 1) also suggested DIII seemed to play a necessary role in the interaction between insect sodium channel and BmK IT2.

### Residues in DIII SS2-S6 critical for the sensitivity of DmNa_v_1 to BmK IT2

**Figure 4:**
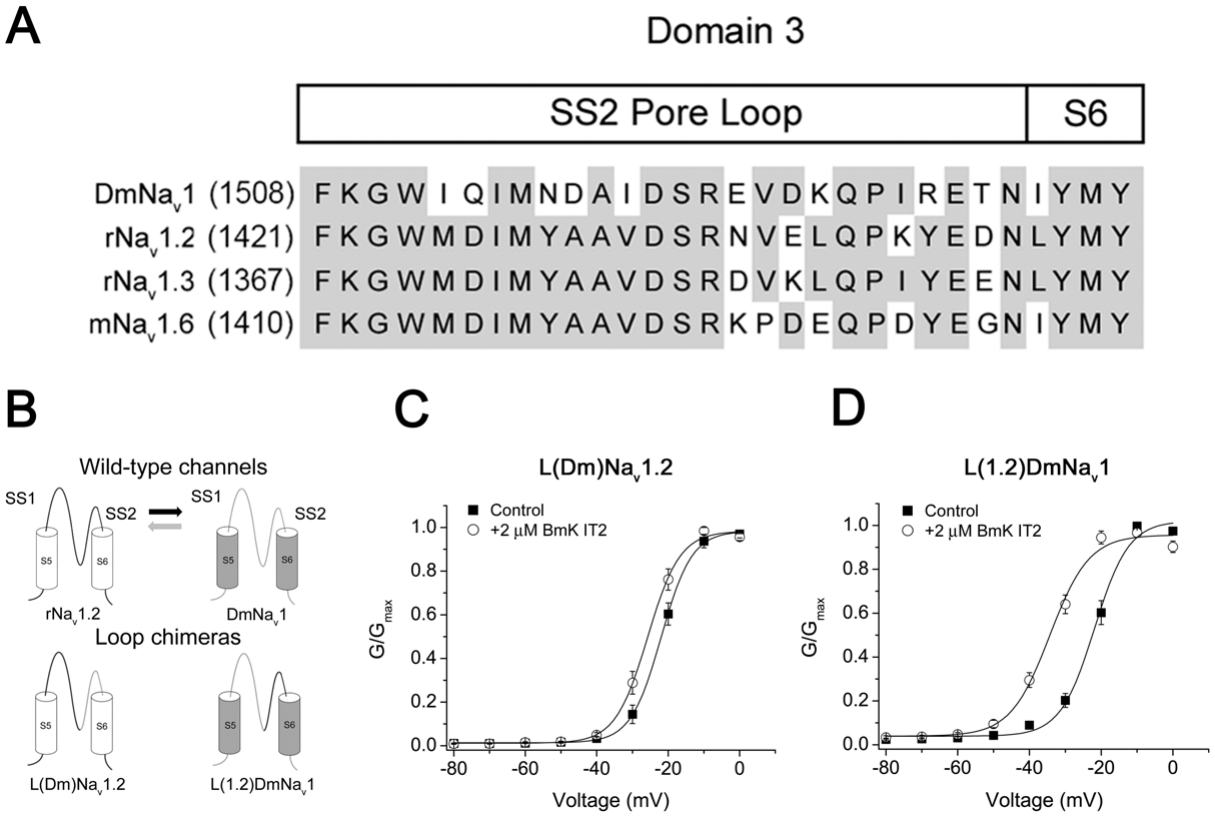
Analysis of DmNa_v_1-Na_v_1.2 DIII SS2 loop chimeras. A. Sequences of SS2 loop in DIII of wild-type VGSCs. B. Diagram illustrating the composition of the SS2 loop chimeras L(Dm)Na_v_1.2 and L(1.2)DmNa_v_1 (Na_v_1.2 SS2 loop: grey; DmNa_v_1 SS2 loop: black). C–D. Effect of BmK IT2 on voltage-dependent activation of L(Dm)Na_v_1.2 and L(1.2)DmNa_v_1 with a prepulse (PP) of −10 mV for 25 ms. ▪, control conditions; ○, 2 µM BmK IT2.

To further clarify the possible interaction site in DIII, a series of mutations have been perfomed in DIII SS2-S6 pore loops of rNa_v_1.2α and DmNa_v_1. The mutagenesis design was based on the previous report that suggested DIII SS2-S6 might be involved in the binding of LqhIT2 [11]. First, to verify whether this region accounted for BmK IT2 binding, the DIII SS2 loops were compared (Fig. 4A) and exchanged between DmNa_v_1 and rNa_v_1.2 (Fig. 4B), giving rise to two loop chimeras: L(Dm)Na_v_1.2 and L(1.2)DmNa_v_1. Unexpectedly, the whole loop replacement in DmNa_v_1 (I_1512_ to I_1534_) by that of rNa_v_1.2 (M_1425_ to L_1447_) resulted in channels hardly expressed in *Xenopus* oocytes even accompanied by TipE subunit. Thus for generating robust Na^+^ currents, two residues in rNa_v_1.2-type loop had to be restored as present in DmNa_v_1 (I_1529_/R_1530_) (See *Material and Methods*). Double mutant DmI_1529_K/R_1530_Y was then produced as the compensation of the incomplete loop substitution.

Similar to the case of chimera ChD3, in the presence of 2 µM BmK IT2 and a 25 ms prepulse, the voltage-dependent activation of L(Dm)Na_v_1.2 displayed a mild but significant shift with ΔV_1/2_ of about −5 mV (p<0.05, n = 8, Fig. 4C and Table 2). As for L(1.2)DmNa_v_1 (Fig. 4D), the substitution by most part of the DIII SS2 loop from rNa_v_1.2 could not prevent BmK IT2-induced shift in the voltage of half-maximal activation (ΔV_1/2_ = −12.48 mV). Interestingly, however, unlike wild type DmNa_v_1, the slope factor of its activation curve was barely affected by BmK IT2 (L(1.2)DmNa_v_1: Δ*k_m_*<1 mV, n = 8; DmNa_v_1: Δ*k*_m_ = +4.25 mV, n = 10; Table 2). It was noticeable that double-mutant DmI_1529_K/R_1530_Y exhibited largely attenuated sensitivity to 2 µM BmK IT2 as the toxin-induced ΔV_1/2_ decreased to −5.06 mV with the slope factor (*k*_m_) unchanged (Table 2). The results indicats that the DIII SS2-S6 pore-loop of DmNa_v_1 plays a major role in BmK IT2 interaction and it was the main contributor in conferring BmK IT2 sensitivity to rNa_v_1.2.

**Figure 5:**
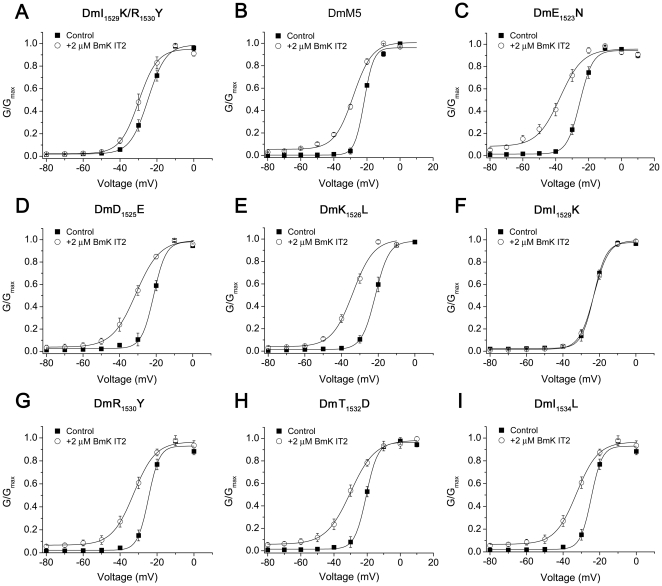
Site-directed mutations introduced in DIII SS2-S6 loop of DmNa_v_1. A–I. Normalized conductance-voltage curves for the indicated mutant channels before (▪) and after (○) application of 2 µM BmK IT2, with a prepulse (+PP) in all cases.

To determine the residue(s) in this region critical for the interaction with BmK IT2, a series of site-directed mutations of DmNa_v_1 were produced (see *Materials and Methods*). All mutants displayed gating parameters (Table 2) similar to those of wild type DmNa_v_1, ruling out the possibility that the alteration of gating behavior was involved in variation of BmK IT2 sensitivity. Subsequent analysis demonstrated that among all the mutants (Fig. 5), the potency of 2 µM BmK IT2 was obviously decreased on DmM5, DmI_1529_K, DmR_1530_Y and DmI_1529_K/R_1530_Y, with respect to wild-type DmNa_v_1. The alterations in voltage-dependent activation induced by 2 µM BmK IT2 were in the order that (ΔV_1/2_, Δ*k*_m_): DmNa_v_1 (−11.92 mV, +4.25 mV)>DmR_1530_Y (−5.11 mV, +3.66 mV), DmM5 (−6.56 mV, +2.88 mV)>DmI_1529_K/R_1530_Y (−5.06 mV, +0.20 mV)>DmI_1529_K (+0.12 mV, +0.60 mV). Notably, mutant DmI_1529_K was less sensitive to BmK IT2 (EC_50_ = 15.6±3.60 nM, Talbe 1), indicating an especially critical role of residue I_1529_ in the interaction with BmK IT2.

## Discussion

### VGSC subtype-selectivity of BmK IT2

BmK IT2 was classified into the group of β-depressant insect toxin because: 1) it shares high sequence similarity with other well-defined depressant anti-insect toxins, such as LqhIT2, LqqIT2 and BjIT2 [26]; 2) BmK IT2 is toxic to insect but not mammals [27], [28]. This insect-selectivity was also observed in binding experiments tested on cockroach nerve cords which displayed a 200–300 fold higher affinity with BmK IT2 than rat brain synaptosomes [17]. However, like some other depressant β-toxins [21], [22], [23], BmK IT2 also evolves function against mammals, e.g. antinociceptive and anticonvulsant activities in rat models [19]. As recent studies have mostly focused on the pharmacological phenotype of BmK IT2, the underlying mechanism and molecular target in rat brain remain unintelligible. In this study, the efficacay and selectivity of BmK IT2 was assayed for the first time among independently cloned VGSCs from insect (DmNa_v_1) and mammalian central nervous system (i.e. rNa_v_1.2, rNa_v_1.3 and mNa_v_1.6) expressed in *Xenopus* oocytes.

Results showed that the main effects of BmK IT2 on DmNa_v_1 included a decrease of peak Na^+^ current (by ∼20%) and a significant hyperpolarizing shift of the activation. These are typical effects for scorpion depressant β-toxins. The increase of the slope value of activation curve, reflecting the decreased voltage dependence of activation process and a larger subthreshold channel open probability, is also observed in previous reports characterizing the function of depressant β-toxins LqqIT2 and LqhIT2 [29], [30].

In contrast, three mammalian VGSCs were totally insensitive to BmK IT2. The low affinity of BmK IT2 to rat brain synaptosomes can be explained by the insensitivities of Na_v_1.2 and Na_v_1.6,which are dominant VGSC subtypes spreading throughout CNS [31], [32], to BmK IT2. It is noteworthy that BmK IT2 was capable of inhibiting the total Na^+^ currents in rat dorsal root ganglion (DRG) neurons [20]. According to our results that Na_v_1.2, Na_v_1.3 and Na_v_1.6 are BmK IT2-insensitive, the action of BmK IT2 on Na^+^ currents of DRG neurons may be a result of selective modulation on other neuronal VGSC subtypes, most likely Na_v_1.7, Na_v_1.8 and/or Na_v_1.9 channels. Thus, it may allow us to speculate that peripheral nerve VGSC subtypes might be the major targets responsible for BmK IT2-induced anti-nociception and anti-convulsant effects in rat models, though the subtypes or other membrane proteins that are possibly involved in the working mechanism of BmK IT2 still need to be further characterized.

### Construction of insect-mammalian chimeric channels

Since the insect and mammalian VGSCs are highly similar in both structural and functional properties, insect-mammalian chimeras could be constructed to determine the regions responsible for the toxin recognition and interaction. Previously for localizing the insect VGSC domain that binds β-excitatory toxin AahIT, a chimeric channel was constructed from rat brain rNa_v_1.2 in which DII was replaced by that of *Drosophila* [12]. Here we also chose rNa_v_1.2 as backbone of chimeric channels that accepted insect VGSC domains considering that: 1) rNa_v_1.2 channel is insensitive to BmK IT2 at very high concentration (e.g. 20 µM); 2) as a typical VGSC subtype from mammalian nervous system, rNa_v_1.2 channel has been well characterized in *Xenopus* oocytes and displays an excellent performance in expression level. The four resulting insect-mammalian chimeras were all expressed functionally and identified to be TTX-sensitive VGSCs. Chimeric channels could be regulated by β1 subunit except ChD4 that seemed insensitive to either β1 or TipE subunit. The low current density of ChD4 was improved only by prolonging the expression time duration. These results agreed with the finding that the binding site for β1 was localized to DIV in rNa_v_1.2 [33] and implicated that TipE might not regulate DmNa_v_1 through DIV.

To directly reveal the BmK IT2 binding region(s) in DmNa_v_1 channel and confirm the results obtained in Na_v_1.2 backbone chimeras, we also attempted to generate the mammalian-insect chimeras in which the independent domains of DmNa_v_1 were replaced by those of rNa_v_1.2. Unfortunately, due to the rather low expression level, these chimeras failed to serve as satisfying candidates for the subsequent pharmacological analysis.

### The binding feature of BmK IT2 on DmNa_v_1

The classical voltage-sensor trapping model indicates that β-toxins function as a stablizer of activated state of VGSCs by trapping the outward DII S4 and hereby shift the activation threshold to more hyperpolarized potentials [4].

**Figure 6:**
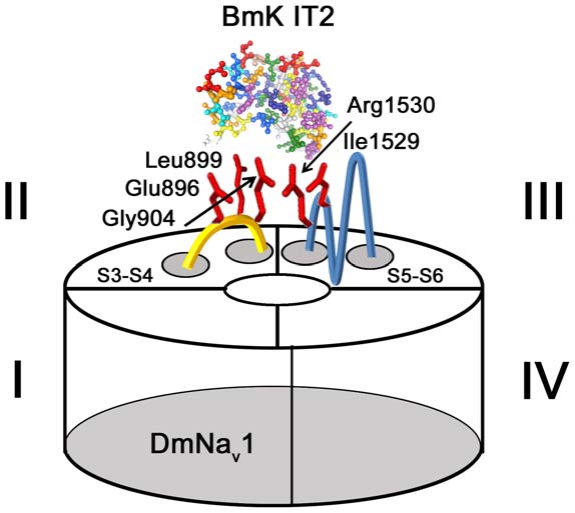
Schematic presentation of domain arrangement and key residues involved in BmK IT2-DmNa_v_1 interaction. Schematic BmK IT2 structural model (in amino residue) was constructed by Swiss-model Workspace (http://swissmodel.expasy.org) based on the known structure of the depressant β-toxin LqhIT2 (>80% similarity in sequence) (PDB accession 2i61A). Key residues involved in BmK IT2 interaction with DmNa_v_1 were highlighted in red and indicated with sequence numbers on extracellular loop of DII (yellow) and DIII (blue).

In this study, mutations of G_904_, E_896_, L_899_ in DII S4 of DmNa_v_1 completely abolished the action of BmK IT2, suggesting that, like other β-toxins (e.g. CssIV and Cn2), BmK IT2 functionally interacts with DmNa_v_1 through DII S4 as described in the voltage-sensor trapping model (Fig. 6). However, these residues could not serve as a major determinant to BmK IT2 sensitivity as they are well conserved in the BmK IT2-insensitive mammalian channels like rNa_v_1.2, rNa_v_1.3 and mNa_v_1.6. The subsequent study revealed that DIII rather than DII could confer BmK IT2 insect-preference to mammalian sodium channel. The channel epitope that interacts with BmK IT2 was further narrowed down to residues around the N-part of DIII SS2-S6 loop (I_1512_/Q_1513_/N_1516_/D_1517_/I_1519_) as well as the hydrophobic I_1529_ and the positive R_1530_, implying the hydrophobic and electrostatic interactions may both be decisive for toxin binding. Although the residue alterations at positions 1512–1526 and at position 1530 had minor impact on toxin efficacy, the exchange of hydrophobic Ile at position 1529 in DmNa_v_1 to the Lys present in rNa_v_1.2 largely impaired the toxin-channel interaction. Thus the central role of I_1529_ seemed to support the hydrophobic interaction in toxin-channel inter-recognition (Fig. 6).

Apparently, the receptor site for BmK IT2 involves at least two channel regions: 1) DII S3–S4 linker, for mediating toxin functional interaction with voltage-sensor; 2) DIII SS2 loop: the determinant for BmK IT2 specific targeting.This is different from the case for excitatory β-toxin: the receptor site for AahIT was found to reside mainly in DmNa_v_1 DII [12]. Our result confirms that the receptor sites for excitatory and depressant β-toxins are not identical on insect VGSC [10], [34], however, they have an overlapping region, i.e. DII. That is in concordance with the fact that excitatory toxins can compete with depressant toxins for the high-affinity binding site on insect nerve membrane [11], [34].

Interestingly, despite targeting VGSCs from different phyla, the binding features of BmK IT2 and Tz1, a β-like toxin that can strongly affect the activation of muscular Na_v_ channel but was incapable of affecting the activation of cardiac and peripheral nerve Na_v_ channels [5], appear very similar: toxins recognize and bind to the pore loop of DIII and then are capable of trapping the outward movement of voltage-sensor in DII, thus lowering the threshold for channel activation.

### Conclusion

The insect-selectivity of BmK IT2 was highlighted in this study when differentiating between heterologously expressed VGSC subtypes from insect and mammalian central nervous system. The results suggested Na_v_1.2, Na_v_1.3, and Na_v_1.6 channels were not involved in mediating the BmK IT2-induced antinociceptive and anticonvulsant effect in rat models. The study revealed the receptor site on insect VGSC DmNa_v_1 for depressant β-toxin BmK IT2 consisted of at least two regions, i.e. DII and DIII. The recognition epitope for insect-preference were localized to the hydrophobic residues within DIII pore-loop SS2-S6. Finally, the inter-species chimeric channels employed here may provide a promising operation for identifying putative binding site(s) in VGSCs targeted by other specific modulators.

## Supporting Information

Figure S1 Sequences of the DmNa_v_1 mutants indicating the mutated residues in DII and DIII. The loop chimera L(Dm)Na_v_1.2 was produced by replacing the diversed residues within DIII SS2-S6 loop of rNa_v_1.2 by those from DmNa_v_1 (underlined residues) correspondingly. In addition, single- or multiple-mutagenesis were also employed on DmNav1, giving rise to the loop-chimera or mutants listed below. Black dots in loop-chimera/mutants indicated the unchanged residues compared to the sequence of L(Dm)Na_v_1.2 (or DmNa_v_1).
(1.80 MB TIF)

Figure S2 Effect of BmK IT2 on mammalian wild-type VGSCs. Normalized conductance-voltage (G-V) curves of rNa_v_1.2, rNa_v_1.3, mNa_v_1.6 in absence (▪) and presence of 20 µM (○) and 50 µM (△) BmK IT2, with a 25 ms prepulse.
(1.34 MB TIF)

Figure S3 Dose-response curves for effects of BmK IT2 at DmNa_v_1 and indicated mutants. The EC_50_ values were determined by measuring the currents induced by the toxin at a test pulse of −40 mV (Table 1). The protocol used are shown in the inset.
(5.03 MB TIF)

Table S1 The localizations of mutated bases are underlined in nucleotide sequence of all the primers. For loop chimeras, the deduced amino acid residues of mutated positions are indicated beneath.
(0.07 MB DOC)
