# Supplementary figures and images for: Localization of Receptor Site on Insect Sodium Channel for Depressant β-toxin BmK IT2
Source: PLoS One. 2011 Jan 14;6(1):e14510. doi: 10.1371/journal.pone.0014510 (PMC3021515; doi:10.1371/journal.pone.0014510)

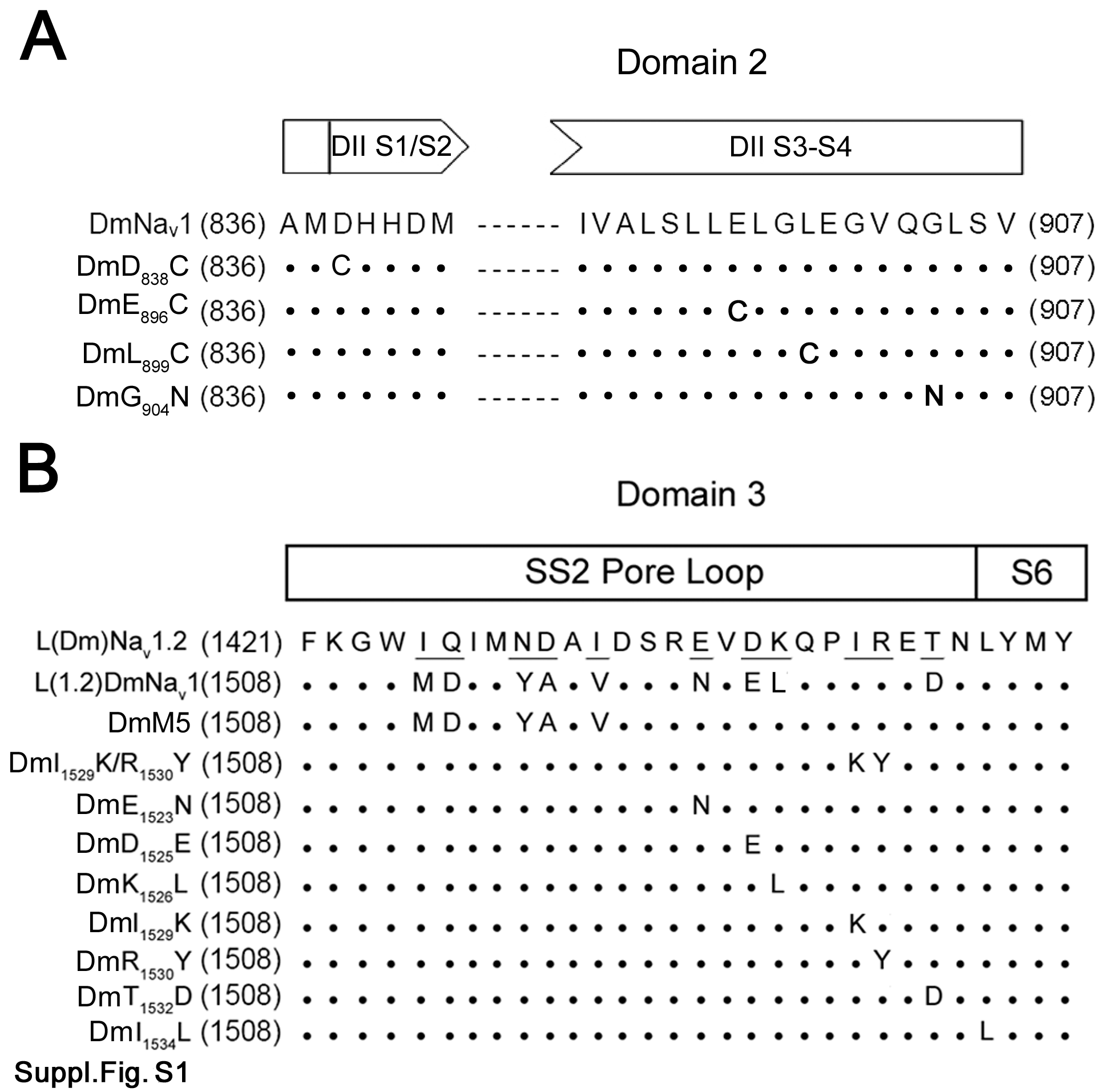

Supplement: Figure S1 — Sequences of the DmNav1 mutants indicating the mutated residues in DII and DIII. The loop chimera L(Dm)Nav1.2 was produced by replacing the diversed residues within DIII SS2-S6 loop of rNav1.2 by those from DmNav1 (underlined residues) correspondingly. In addition, single- or multiple-mutagenesis were also employed on DmNav1, giving rise to the loop-chimera or mutants listed below. Black dots in loop-chimera/mutants indicated the unchanged residues compared to the sequence of L(Dm)Nav1.2 (or DmNav1). (1.80 MB TIF) [file pone.0014510.s001.tif]

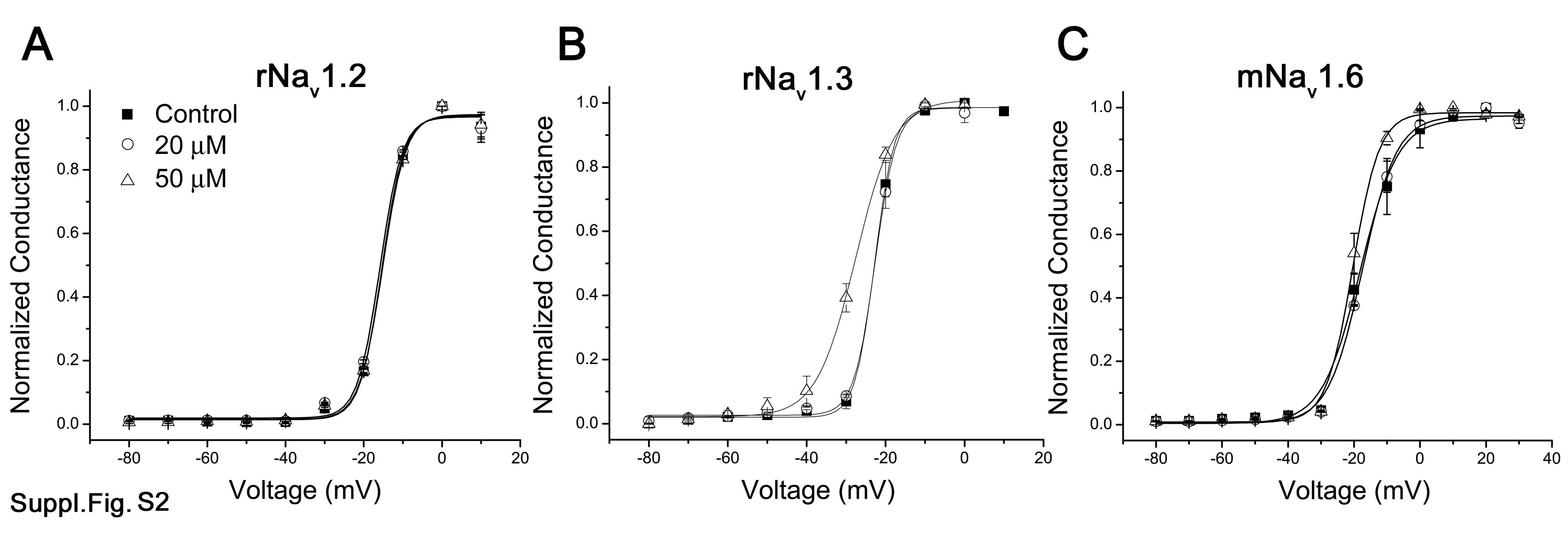

Supplement: Figure S2 — Effect of BmK IT2 on mammalian wild-type VGSCs. Normalized conductance-voltage (G-V) curves of rNav1.2, rNav1.3, mNav1.6 in absence (▪) and presence of 20 µM (○) and 50 µM (△) BmK IT2, with a 25 ms prepulse. (1.34 MB TIF) [file pone.0014510.s002.tif]

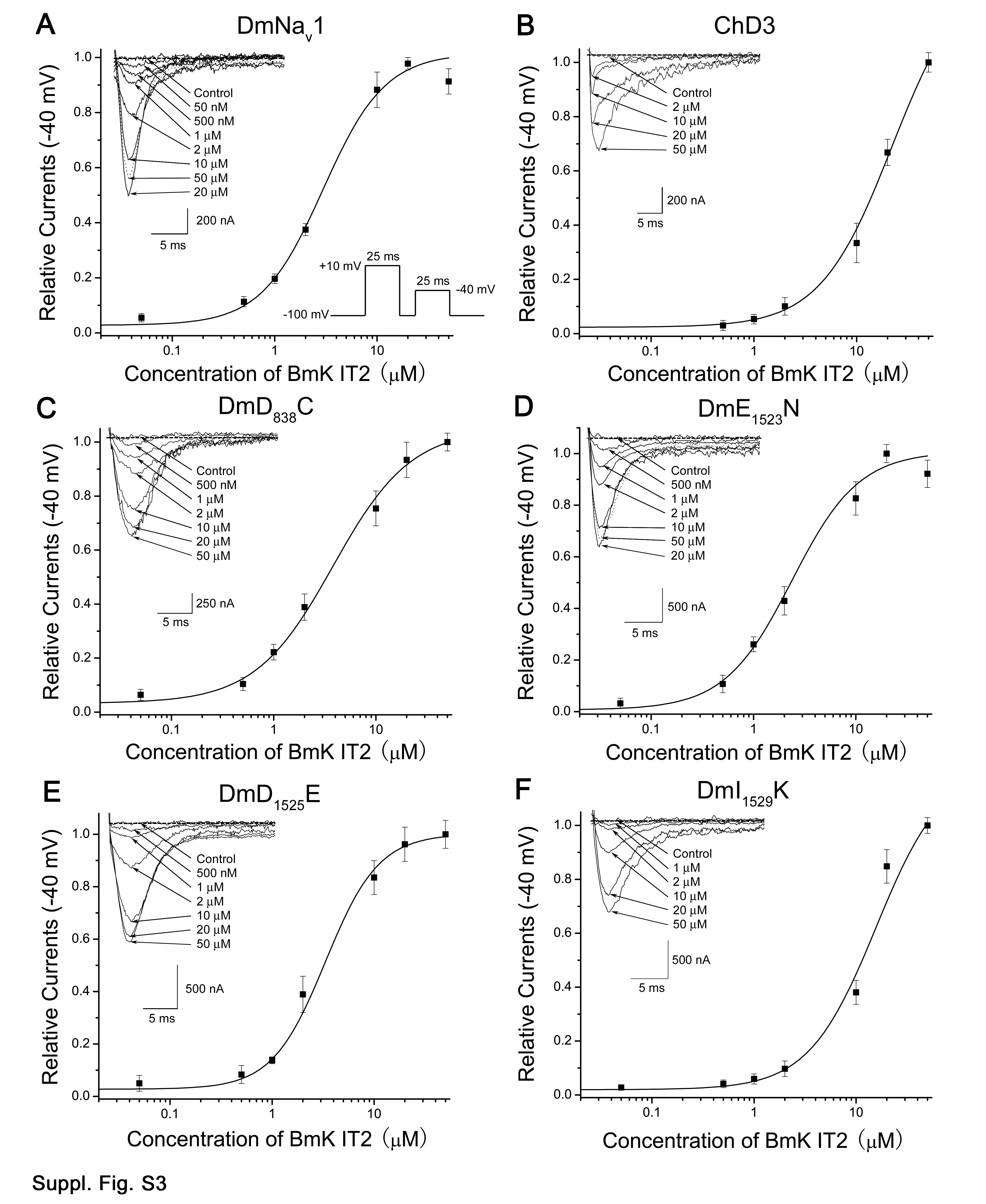

Supplement: Figure S3 — Dose-response curves for effects of BmK IT2 at DmNav1 and indicated mutants. The EC50 values were determined by measuring the currents induced by the toxin at a test pulse of −40 mV (Table 1). The protocol used are shown in the inset. (5.03 MB TIF) [file pone.0014510.s003.tif]
