# Supplementary material for: Localization of Receptor Site on Insect Sodium Channel for Depressant β-toxin BmK IT2
Source: PLoS One. 2011 Jan 14;6(1):e14510. doi: 10.1371/journal.pone.0014510 (PMC3021515; doi:10.1371/journal.pone.0014510)
Supplement: Table S1 — The localizations of mutated bases are underlined in nucleotide sequence of all the primers. For loop chimeras, the deduced amino acid residues of mutated positions are indicated beneath. (0.07 MB DOC) [file pone.0014510.s004.doc]

**Table S1: Sequences of forward and reverse primers used in construction of domain/loop chimeras and site-directed mutants.**

| Mutant Name | Primer Name* | Nucleotide sequence/Mutated residues | Position |
| --- | --- | --- | --- |
| Domain Chimeras: | | | |
| ChD1 | S4 (+XhoI) | 5’-CGAATTAACTCGAGCTCGGTACCC | N-teminal |
|  | A443(-XhoI) | 5’-TCGATATCCTCCAGAGGAGTGGA | N-teminal |
|  | S421(-XhoI) | 5’-TCCACTCCTCTGGAGGATATCGA | N-teminal |
|  | A2017(+XmaI) | 5’-TATACGATACCCGGGACTGATGC | DI-DII |
| ChD2 | S1999 (+XmaI) | 5’-GCCTCCCGGGGGTCGTATACCTCACATGGCGATCTA | DI-DII |
|  | A3498 (+BglII) | 5’-GGACAGATCTTCCAGTTGCGTCTGCTCCTTGATCCC | DII-DIII |
| ChD3 | S3486 (+BglII) | 5’-TGGAAGATCTCATCGGGGATGGCATGG | DII-DIII |
|  | A4944 (+BstEII) | 5’-GGTCATGGTGACCATGTTCAGACCAATG | DIII-DIV |
| ChD4 | S4926 (+BstEII) | 5’-GAACATGGTCACCATGACCCTC | DIII-DIV |
|  | A6454 (+PacI) | 5’-GTGGTAACTTAATTAAGCTCCTGGCTC | C-teminal |
| Loop Chimeras: | | | |
| L(Dm)Na_v_1.2 | A8721 | 5’-AATAGCATCGTTCATGATCTGTATCCATCC  I D N Q I | DIII SS2-S6 |
|  | S8722 | 5’-GACTCAAGAGAGGTAGACAAGCAGCCC  D K | DIII SS2-S6 |
|  | A8743 | 5’-GCAGTTCCACCTCTCTTGAGTCAACAG  D E | DIII SS2-S6 |
|  | S8744 | 5’-AGCCCATACGCGAAACCAATCTC  I R T | DIII SS2-S6 |
| L(1.2)DmNa_v_1 | A4575 | 5’-GATATCCATCCAGCCTTTGAAGGTG  D M | DIII SS2-S6 |
|  | S4576 | 5’-ATGTACGCTGCTGTCGATTCACG  Y A V | DIII SS2-S6 |
|  | A4610 | 5’-AGCTCCACATTTCGTGAATCGACAG  L E N V | DIII SS2-S6 |
|  | S4611 | 5’-GCAGCCAAAGTATGAAGACAACATC  D | DIII SS2-S6 |
| Site-directed Mutants: | | | |
| DmM5 | A4575 | 5’-GATATCCATCCAGCCTTTGAAGGTG  D M | DIII SS2-S6 |
|  | S4576 | 5’-ATGTACGCTGCTGTCGATTCACG  Y A V | DIII SS2-S6 |
| DmD_838_C | S2567DC | 5’-TGGCAATGTGTCACCACGATATG | DII S1-S2 |
|  | A2566DC | 5’-TGAACATCGTGTTGACCACAATGC | DII S3 |
| DmE_896_C | S2765 | 5’-GTGTCCAGGGTCTGTCCGTATT | DII S3 |
|  | A2764EC | 5’-CCTCGAGTCCCAGGCACAATAG | DII S3 |
| DmL_899_C | S2765 | 5’-GTGTCCAGGGTCTGTCCGTATT | DII S3 |
|  | A2764LC | 5’-CCTCACATCCCAGTTCCAATAGC | DII S3 |
| DmG_904_N | S2759GN | 5’-TCGAGGGTGTCCAGAATCTGTCC | DII S3-S4 |
|  | A2758GN | 5’-GTCCCAGTTCCAATAGCGATAGGG | DII S3-S4 |
| DmI_1529_K/R_1530_Y | S4611KY | 5’-GCAACCAAAGTATGAAACGAACATC | DIII SS2-S6 |
|  | A4610 | 5’-TTGTCCACCTCTCGTGAATCGATAG | DIII SS2-S6 |
| DmE_1523_N | S4607EN | 5’-ACAAGCAACCAATTCGTGAAACG | DIII SS2-S6 |
|  | A4606EN | 5’-CCACGTTTCGTGAATCGATAGCATC | DIII SS2-S6 |
| DmD_1525_E | S1525DE | 5’-CAACCAATTCGTGAAACGAACATC | DIII SS2-S6 |
|  | A1525DE | 5’-CTTCTCCACCTCTCGTGAATCGA | DIII SS2-S6 |
| DmK_1526_L | S4598KL | 5’-AGAGGTGGACCTGCAACCAATTCG | DIII SS2-S6 |
|  | A4598KL | 5’-CGTGAATCGATAGCATCGTTCATG | DIII SS2-S6 |
| DmI_1529_K | S4611IK | 5’-GCAACCAAAACGTGAAACGAACATC | DIII SS2-S6 |
|  | A4610 | 5’-TTGTCCACCTCTCGTGAATCGATAG | DIII SS2-S6 |
| DmR_1530_Y | S4611RY | 5’-GCAACCAATTTATGAAACGAACATC | DIII SS2-S6 |
|  | A4610 | 5’-TTGTCCACCTCTCGTGAATCGATAG | DIII SS2-S6 |
| DmT_1532_D | S1532TD | 5’-CCAATTCGTGAAGACAACATCTACATG | DIII SS2-S6 |
|  | A1532 | 5’-TTGCTTGTCCACCTCTCGTGAATC | DIII SS2-S6 |
| DmI_1534_L | S1534IL | 5’-CCAATTCGTGAAACGAACCTCTACATG | DIII S6 |
|  | A1532 | 5’-TTGCTTGTCCACCTCTCGTGAATC | DIII S6 |

*(S: sense; A:anti-sense)
